# Adverse events in gynaecological radiotherapy: Better Reporting, Interpretation and Dissemination to Generate Evidence (BRIDGE)^☆^

**DOI:** 10.1016/j.tipsro.2026.100439

**Published:** 2026-09-27

**Authors:** Sofia Spampinato, Ewa Burchardt, Magdalena Stankiewicz, Amelia Barcellini, Gemma Eminowicz, Barbara Šegedin, Margit Valgma, Kari Tanderup, Remi Nout, Kathrin Kirchheiner

**Affiliations:** a Department of Radiotherapy, Erasmus MC Cancer Institute, University Medical Center Rotterdam, Rotterdam, the Netherlands; b Department of Radiotherapy and Gynaecological Oncology, Greater Poland Cancer Center in Poznan, 61-866 Poznań, Poland; c Electroradiology Department, Poznan University of Medical Sciences, 61-701 Poznań, Poland; d Brachytherapy Department, Maria Sklodowska-Curie National Research Institute of Oncology, Gliwice Branch, Gliwice, Poland; e Department of Internal Medicine and Medical Therapy, University of Pavia, Pavia, Italy; f Radiation Oncology Unit, Clinical Department, National Center for Oncological Hadrontherapy (CNAO), Pavia, Italy; g Department of Oncology, University College London Hospital, London, United Kingdom; h Department of Radiotherapy, Institute of Oncology, Ljubljana, Slovenia; i Faculty of Medicine, University of Ljubljana, Ljubljana, Slovenia; j Radiotherapy Centre, North Estonia Medical Centre Foundation, Tallinn, Estonia; k Danish Centre for Particle Therapy, Aarhus University Hospital, Aarhus, Denmark; l Department of Clinical Medicine, Aarhus University, Aarhus, Denmark; m Department of Radiation Oncology, Comprehensive Cancer Center, Medical University of Vienna, Vienna, Austria

## Abstract

Accurate adverse events (AE) reporting is essential in gynaecological radiotherapy, as multimodal approaches become increasingly common. While these strategies improve outcomes, they may increase the risk of complications affecting patients' quality of life. The BRIDGE (Better Reporting, Interpretation and Dissemination to Generate Evidence) initiative aims to improve consistency and understanding in AE assessment across the radiotherapy community. This analysis investigated whether interpretation of the same clinical information leads to variability in AE grading using the Common Terminology Criteria for Adverse Events (CTCAE) v6.0. An interactive poll conducted during an ESTRO webinar used two clinical scenarios. Among 71 attendees, 30 responded to the poll and 23 consented to share their data. Variation in severity scoring was observed, especially for subjective and lower-grade AEs, whereas higher agreement occurred when clinical details aligned with CTCAE terminology. Findings highlight the importance of training, clear clinical context, and integration of patient-reported outcomes to support person-centred care.

## Highlights

• BRIDGE initiative promotes consistent AE reporting in gynaecological radiotherapy.
• CTCAE v6.0 AE grading variability was assessed via live poll using clinical scenarios.
• Subjective, lower grade AEs showed highest scoring inconsistency among responders.
• Training and clinical context clarify CTCAE grading criteria, improving consistency.
• Use of patient-reported outcomes improves detection and grading of symptomatic AEs.

## Introduction

Management of gynaecological cancers has undergone a transformative period marked by greater personalisation of care and the integration of multiple treatment modalities. Radiotherapy continues to play a central role, particularly in cervical and endometrial cancers, while recent evidence from clinical trials supports its combination with systemic therapies, including chemotherapy, immunotherapy, and targeted agents [1], [2]. In locally advanced cervical cancer, the addition of immunotherapy to standard chemoradiotherapy and the use of induction chemotherapy have been associated with improved survival outcomes [3], [4]. In endometrial cancer, combined chemoradiotherapy has also been shown to improve survival in selected high-risk patients [5].

Treatment intensification through multimodality approaches can improve survival outcomes, but may also carry a risk of increased adverse events (AEs). These events can compromise treatment adherence and consequently reduce its efficacy, especially for regimes combining multiple modalities and agents with a prolonged duration [6], [7]. In addition, when side effects persist after treatment, they can negatively affect daily functioning and long-term quality of life (QoL). Therefore, AE reporting is essential for understanding the benefit-risk balance of treatment strategies and for guiding clinical decision-making and effective consent-taking [8]. However, this process remains complex and multi-step, and information can be lost or distorted as it moves from patient experience to the final reporting in publications [9].

Given the increasing complexity of AE reporting in evolving clinical trials, the use of a standardized and consistent language is essential to ensure the interpretation and comparability of results. The Common Terminology Criteria for Adverse Events (CTCAE), developed by the US National Cancer Institute (NCI), is recognised as the standard system to report AEs in oncology clinical trials [10]. This terminology includes unique AE *“terms”* mapped to the Medical Dictionary for Regulatory Activities (MedDRA) and categorized in three groups: laboratory-based, observable or measurable, and symptomatic [11]. Severity grades (from 1 to 5) are assigned based on the impact of the AE on clinical management, activities of daily living (ADL), dose modifications, or treatment discontinuation [12].

Over time, CTCAE has undergone continuous evolution with the introduction of new AEs for a more comprehensive reporting better reflecting therapeutic advances. In addition, some definitions of AEs terms and severity grades have also been revised to more accurately capture observations from clinical practice. The most recent version (v6.0), released in July 2025, includes 850 AEs compared with 837 in v5.0. Updates involve the addition of new AEs, as well as the removal of some terms and modifications to severity grade definitions. Such changes, if not carefully accounted for, may complicate correct assessment, increase inter-rater variability, and potentially lead to misinterpretations [13].

Recognising the importance of consistent and accurate AE reporting in clinical trials, members of the European Society of Radiotherapy and Oncology (ESTRO) Gynaecology Focus Group and researchers with expertise in side effects after gynaecological radiotherapy established the BRIDGE (*Better Reporting, Interpretation and Dissemination to Generate Evidence*) initiative. A pilot study compared AEs relevant to gynaecological radiotherapy surveying six radiation oncologists in the team to evaluate clinically relevant changes from CTCAE v3.0 to v5.0. Findings showed that updated severity grade definitions often incorporate less information on clinical observations and interventions, while placing more emphasis on patient-reported symptoms, especially for moderate (grade 2) and severe (grade 3) AEs. These changes affect the interpretation and increase variability in scoring, highlighting the need for improved training and standardisation in AE reporting practices [14].

The release of CTCAE v6.0 provides an excellent opportunity to improve agreement in AE reporting and to further clarify severity grade definitions for clinical trial investigators and the gynaecological radiotherapy community. Building on challenges previously identified in earlier CTCAE versions, this study presents the results of a live polling exercise conducted within the BRIDGE initiative. Participants were asked to grade AEs based on clinical case scenarios. The primary aim was to examine how interpretation of the same clinical information influences AE grading using CTCAE v6.0. The exercise also provided an opportunity to improve understanding of grading criteria and promote consistency in AE reporting across users with varying levels of experience. In addition, the interactive discussion between participants and the BRIDGE panel provided insight into challenges in AE grading and potential approaches to improve consistency in assessment and reporting.

## Material and methods

The live poll was conducted on June 11th, 2026 during a Gyn-SMART (*Gynaecology Series on Modern Applied RadioTherapy*) webinar, an initiative of the ESTRO Gynaecology Focus Group. These webinars focus on practical aspects of gynaecological radiotherapy and are hosted on the ESTRO Hub. Participation is free, and events are promoted through ESTRO social media platforms. The webinar, titled *“Scoring and severity assessment of adverse events in gynaecological radiotherapy: BRIDGE the gap,”* concluded a three-event series dedicated to *“Navigating side effects after gynaecological radiotherapy* [15]*.”*

### Live poll

Participants registered for the webinar through the ESTRO Hub platform and received a Zoom link to access the live session. Following an introductory presentation on CTCAE grading, updates in CTCAE v6.0, common scoring challenges, and the BRIDGE initiative, an interactive poll was conducted using”Slido”, a platform for real-time collection of responses and display of anonymized results. Participants accessed the poll via a QR code displayed during the webinar or through a direct web link. The live poll was managed by the ESTRO staff during the session, and anonymized poll data were subsequently made available for this analysis.

### Consent for data sharing

Upon accessing Slido, participants were asked to provide consent for the use of their anonymized responses in offline research analyses. Responses from participants who declined consent were displayed only during the live poll and excluded from this analysis. Participants were also asked to provide information regarding their professional background and experience with CTCAE scoring. All data were collected and analysed anonymously, with no identifiable personal information recorded or linked to responses. As the activity involved anonymized data collected during the webinar, formal ethical approval was not required.

### Clinical cases

Three radiation oncologists (AB,EB,MS) identified clinical cases for the scoring exercise, which were subsequently reviewed and discussed within the team. Two cases were chosen based on their complexity, relevance to updates introduced in CTCAE v6.0, and their potential to stimulate discussion among participants. Two radiation oncologists (EB,MS) prepared the cases for presentation including relevant clinical and treatment information, timing of AE assessment, and details on symptoms evolution and interventions (Supplementary Material). At the end of each case presentation, participants were asked to score selected AEs, with severity grade definitions displayed during the poll to facilitate interpretation. At the end of the scoring, results were presented in real-time and discussed with the BRIDGE panel (BS,EB,GE,KK,KT,MS,SS).

## Results

A total of 183 users registered for the webinar, 71 of whom participated live. Among attendees, 30 responded to the poll and 23 authorized data sharing for research (Table 1). Respondents were primarily radiation oncologists (*n* = 17), followed by radiation therapists (*n* = 3), and most reported CTCAE scoring experience (*n* = 19).

**Table 1:** Interactive poll participants overview.

**Profession**	**n (%)**
*Radiation Oncologist*	17 (73.9%)
*Radiation Therapist*	3 (13.0%)
*Medical Physicist*	1 (4.3%)
*Other (Researcher)*	2 (8.7%)
**Professional experience (years)**	
*<5*	7 (30.4%)
*5 to 10*	2 (8.7%)
*>10*	14 (60.9%)
**Experience with CTCAE scoring**	
*Yes*	19 (82.6%)
*No*	4 (17.4%)

Case 1 included nine AEs to be scored, whereas Case 2 included eight. The complete list of AEs and severity grade definitions, is reported in Table 2.

**Table 2:** List of Adverse Events (AEs) and severity grade definitions included in the live poll.

Adverse Event	Grade 1	Grade 2	Grade 3	Grade 4	Grade 5
*Abdominal pain*	Mild pain	Moderate pain; limiting instrumental ADL or mild/moderate impact on age-appropriate normal daily activity (pediatric)	Severe pain; limiting self-care ADL or severe impact on age-appropriate normal daily activity (pediatric)	–	–
Definition: A disorder characterized by a sensation of marked discomfort in the abdominal region.					
Navigational Note: Record final diagnosis/cause once determined					
*Anorexia^*	Loss of appetite without alteration in eating habits	Oral intake altered without significant weight loss or malnutrition; oral nutritional supplements indicated	Associated with significant weight loss or malnutrition (e.g., inadequate oral caloric and/or fluid intake); tube feeding or TPN indicated	Life-threatening consequences; urgent intervention indicated	Death
*Definition: A disorder characterized by a loss of appetite.*					
*Back pain*	Mild pain	Moderate pain; limiting instrumental ADL or mild/moderate impact on age-appropriate normal daily activity (pediatric)	Severe pain; limiting self-care ADL or severe impact on age-appropriate normal daily activity (pediatric)	–	–
Definition: A disorder characterized by a sensation of marked discomfort in the back region.					
Navigational Note: Consider Injury, poisoning and procedural complications: Infusion related reaction; Record final diagnosis/cause once determined.					
*Bloating*	No change in bowel function or oral intake	Symptomatic, decreased oral intake; change in bowel function	–	–	–
Definition: A disorder characterized by an incomplete paralysis of the muscles of the stomach wall resulting in delayed emptying of the gastric contents into the small intestine.					
*Diarrhea^*	Change in consistency or frequency	Increase of 4–6 stools per day over baseline; moderate increase in ostomy output compared to baseline; limiting instrumental ADL or mild/moderate impact on age-appropriate normal daily activity (pediatric); change in consistency or frequency AND limiting instrumental ADL or mild/moderate impact on age-appropriate normal daily activity (pediatric)	Increase of ≥=7 stools per day over baseline; hospitalization indicated; severe increase in ostomy output compared to baseline; requires IV intervention; limiting self-care ADL or severe impact on age-appropriate normal daily activity (pediatric)	Life-threatening consequences; urgent intervention indicated	Death
Definition: A disorder characterized by an increase in frequency and/or loose or watery bowel movements.					
Navigational Note: Consider Gastrointestinal disorders: Colitis, Enterocolitis.					
*Depression*	Mild depressive symptoms	Moderate depressive symptoms; limiting instrumental ADL or mild/moderate impact on age-appropriate normal daily activity (pediatric)	Severe depressive symptoms; hospitalization not indicated; limiting selfcare ADL or severe impact on age-appropriate normal daily activity (pediatric)	Life-threatening consequences, threats of harm to self or others; hospitalization indicated	Death
Definition: A disorder characterized by melancholic feelings of grief or unhappiness.					
*Fatigue^*	Fatigue relieved by rest	Fatigue not relieved by rest; limiting instrumental ADL or mild/moderate impact on age-appropriate normal daily activity (pediatric)	Fatigue not relieved by rest, limiting self-care ADL or severe impact on age-appropriate normal daily activity (pediatric)	–	–
Definition: A disorder characterized by a state of generalized weakness with a pronounced inability to summon sufficient energy to accomplish daily activities.					
Navigational Note: Record final diagnosis/cause once determined.					
*Flatulence*	Mild symptoms; intervention not indicated	Moderate; persistent; psychosocial sequelae	–	–	–
Definition: A disorder characterized by a discharge of excessive gas from the lower GI tract.					
*Ileus*	Asymptomatic and radiologic observations only	Symptomatic; altered GI function; bowel rest indicated	Severely altered GI function; TPN indicated; tube placement indicated	Life-threatening consequences; urgent intervention indicated	Death
Definition: A disorder characterized by failure of the ileum to transport intestinal contents.					
*Insomnia*	Mild difficulty falling asleep, staying asleep or waking up early	Moderate difficulty falling asleep, staying asleep or waking up early	Severe difficulty in falling asleep, staying asleep or waking up early	–	–
Definition: A disorder characterized by difficulty in falling asleep and/or remaining asleep.					
*Peripheral sensory neuropathy^*	Mild symptoms	Moderate symptoms; limiting instrumental ADL or mild/moderate impact on age-appropriate normal daily activity (pediatric)	Severe symptoms; limiting self-care ADL or severe impact on age-appropriate normal daily activity (pediatric)	Life-threatening consequences; urgent intervention indicated	Death
Definition: A disorder characterized by damage or dysfunction of the peripheral sensory nerves.					
*Urinary tract obstruction^*	Asymptomatic; clinical or diagnostic observations only; intervention not indicated	Symptomatic but no hydronephrosis, sepsis, or renal dysfunction; urethral dilation, urinary or suprapubic catheter indicated	Altered organ function (e.g., hydronephrosis or renal dysfunction); invasive intervention indicated	Life-threatening consequences; urgent intervention indicated	Death
Definition: A disorder characterized by blockage of the normal flow of contents of the urinary tract.					
*Rectal fistula^*	Asymptomatic	Symptomatic, invasive intervention not indicated	Invasive intervention indicated	Life-threatening consequences; urgent intervention indicated	Death
Definition: A disorder characterized by an abnormal communication between the rectum and another organ or anatomic site.					
*Vaginal fistula^*	Asymptomatic	Symptomatic, invasive intervention not indicated	Invasive intervention indicated	Life-threatening consequences; urgent intervention indicated	Death
Definition: A disorder characterized by an abnormal communication between the vagina and another organ or anatomic site.					
*Vaginal stricture^*	Asymptomatic; mild vaginal shortening or narrowing	Vaginal narrowing and/or shortening not interfering with physical examination	Vaginal narrowing and/or shortening interfering with the use of tampons, sexual activity or physical examination	–	–
Definition: A disorder characterized by a narrowing of the vaginal canal.					

*Abbreviations:* ADL = Activities of Daily Living.

Instrumental ADL: preparing meals, shopping for groceries or clothes, using the telephone, managing money, etc.

Self-care ADL: bathing, dressing and undressing, feeding self, using the toilet, taking medications.

*Adverse Event included in Case 1. ^Adverse Event included in Case 2.

The scoring results for Case 1 are presented in Fig. 1. Overall, there was no complete agreement among participants on the severity grading across all AEs. In some instances, agreement was below 70%, with scores distributed across multiple grade options (*Abdominal pain*, *Ileus*, *Insomnia*, *Depression*).

**Fig. 1:**
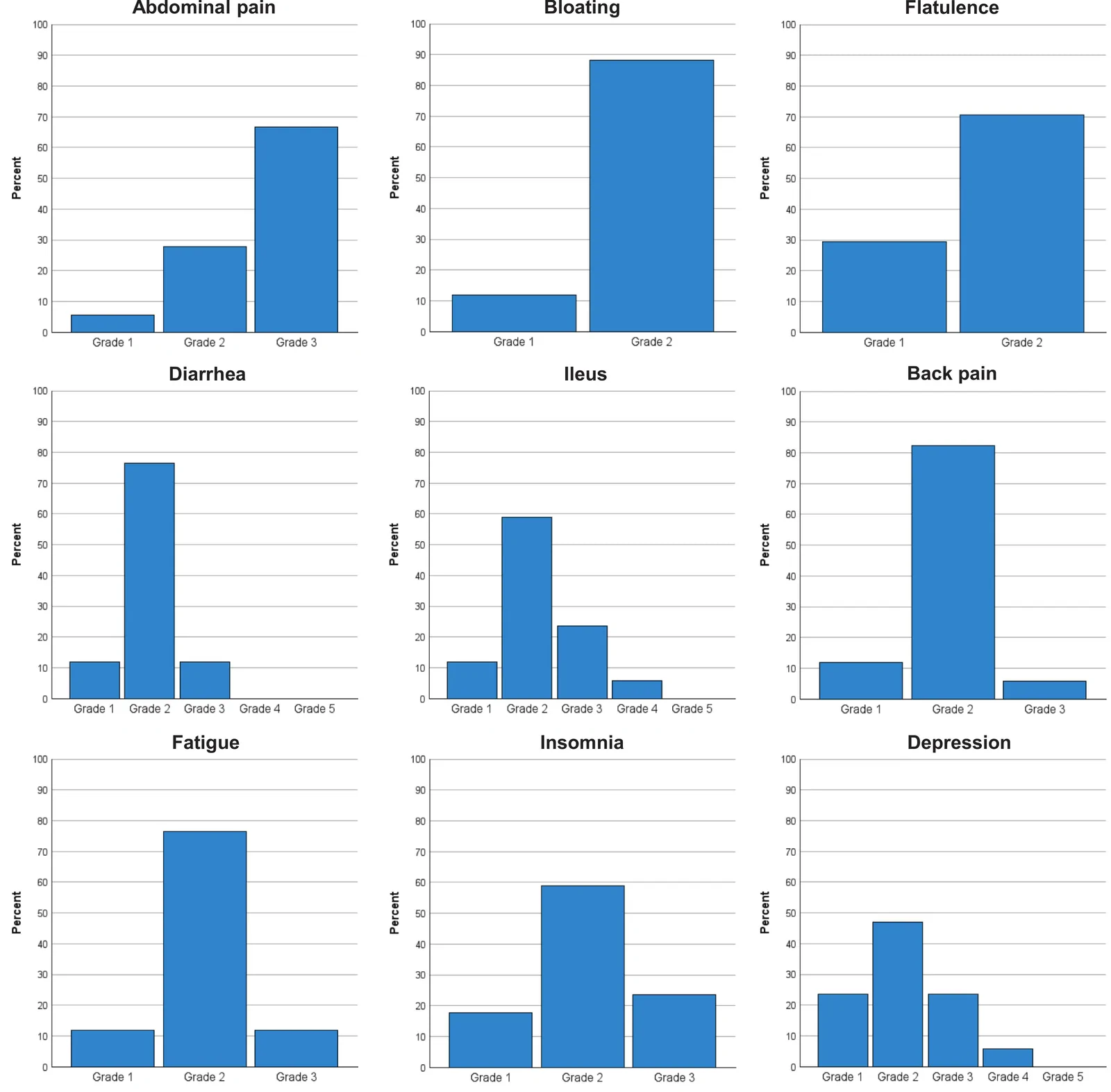
Case 1 Adverse Events scoring results.

For Case 2, complete agreement was observed for *Urinary tract obstruction* (Fig. 2). Overall, higher levels of agreement were recorded compared to Case 1. The main exception was *Vaginal stricture*, for which responses were distributed between grade 2 (36%) and 3 (64%).

**Fig. 2:**
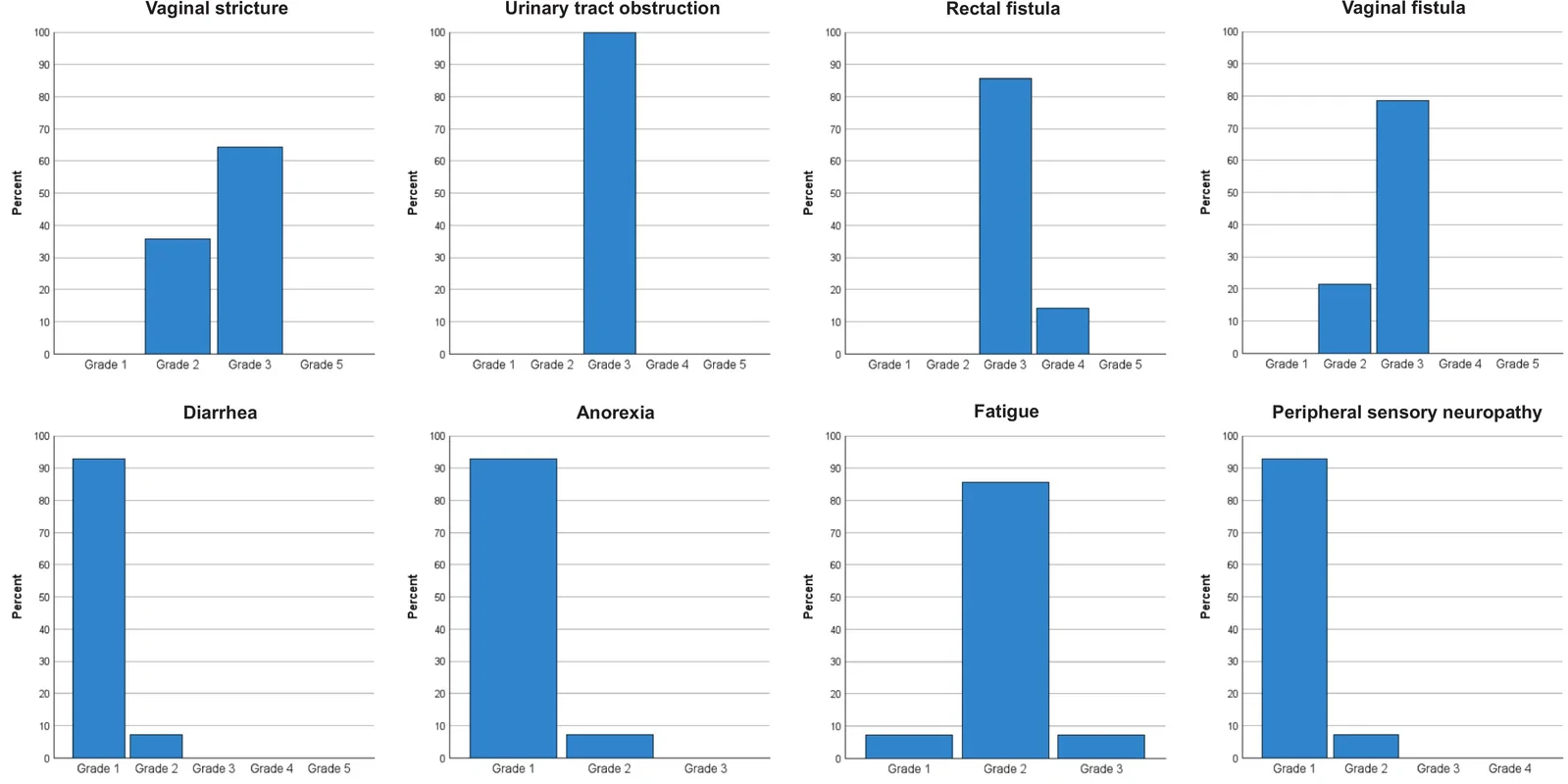
Case 2 Adverse Events scoring results.

The discussion following the scoring exercise highlighted challenges in interpreting symptom burden and its impact on ADLs, and emphasized that comprehensive clinical information and effective clinician–patient communication, particularly the ability to elicit and contextualise subjective symptoms, are essential for improving consistency in AE grading. In addition, the multifactorial nature of AEs, often occurring concurrently and influenced by patient-, disease- and treatment-related factors, further contributed to uncertainty in grading.

Another topic of discussion concerned the appropriate selection and classification of AEs. This was exemplified by the case of *Rectal fistula*, defined as *“a disorder characterized by an abnormal communication between the rectum and another organ or anatomic site*.” In Case 2, the fistula involved rectum and vagina. Therefore, *Rectal fistula* and *Vaginal fistula* would be expected to receive the same grading. However, their interpretation generated uncertainty among respondents, leading to variability in the assigned severity grades, including instances in which *Vaginal fistula* was classified as grade 2 rather than 3 (Fig. 2).

## Discussion

With the increasing adoption of multimodal treatment strategies in gynaecological cancers, accurate AE assessment is essential to evaluate the balance between therapeutic efficacy and risk, and to ensure clear and consistent communication between clinicians and patients. In this context, CTCAE provides a standardized system that facilitates uniform reporting and comparability across clinical trials, while also supporting its use in routine practice for monitoring patients' status and implementing effective management interventions. However, the growing complexity and updates in definitions within newer CTCAE versions may improve granularity but also introduce challenges in interpretation, inter-rater variability, and overall consistency of AE reporting [13]. Recognising these challenges, the BRIDGE initiative was established to streamline different aspects of AE assessment and to promote knowledge sharing through dissemination activities within the radiotherapy community. The present work presents the first results of this effort based on an interactive workshop.

Dedicated training in AE assessment is critical to address many of the challenges highlighted above. Difficulties in interpreting CTCAE definitions and applying severity grades may contribute to AE underreporting and misclassification, while inconsistent use of different versions can lead to discrepancies in reporting [13], [16]. These issues were reflected in the variability observed during the live poll, where differences in interpretation often resulted in inconsistent grading. Despite the widespread use of CTCAE in research and clinical practice, training in AE assessment remains limited, and clinicians often rely on personal experience or informal learning [17]. Structured education activities, including interactive case-based discussions, may offer a valuable opportunity to promote peer-to-peer exchange, facilitating the sharing of experience and learning, and reaching a common understanding of grading criteria. This may help to reducing inter-rater variability and improve consistency, particularly when CTCAE definitions are open to subjective interpretation.

The interactive approach implemented here was inspired by educational and quality assurance activities conducted within the international EMBRACE studies in locally advanced cervical cancer [18]. In this large multi-institutional network, *“Morbidity scoring workshops”* were introduced during annual meetings of the clinical study to address observed variability in AE reporting across participating sites. These workshops involved the presentation of clinical cases, followed by real-time CTCAE scoring and group discussion, aiming to reduce systematic assessment bias and improve consistency among investigators. While EMBRACE workshops often focused on few selected AEs, the BRIDGE cases were intentionally chosen to include multiple concurrent AEs and comprehensive patient information, including symptom evolution, interventions, and prior treatment history. This strategy more closely reflects the complexity of real-world patients presenting with coexisting conditions and highlights the context-dependent nature of AE assessment in clinical practice.

Feedback collected at the end of the interactive exercise indicated that AE grading was perceived as easier when key information on symptoms and interventions was explicitly reiterated during the scoring process. This was reflected in the higher agreement observed in Case 2, where the explicit use and repetition of terms such as “*elective*,” “*non-urgent*,” and “*mild*”, consistent with CTCAE terminology, seemed to facilitate a more straightforward interpretation of severity grades. These findings suggest that clear presentation of clinical details can support more uniform AE assessment. However, translating this approach into routine practice remains challenging, as clinicians must process complex and fragmented information within limited consultation time. This is particularly relevant for symptomatic AEs, for which grading interpretation depends not only on clinical findings and therapeutic interventions, but also on how patients' experiences are elicited and captured by clinicians, and therefore on the ability to ask targeted questions aligned with CTCAE terminology [19], [20].

Literature shows that clinician- and patient-reported symptomatic AEs often differ, with physicians tending to underestimate both the frequency and severity of symptoms compared to patients [9], [21]. This evidence supports the added value of patient-reported outcomes (PROs) to improve symptom detection and better capture the overall treatment burden. In line with this observation, a recent randomized study conducted by EORTC demonstrated that providing clinicians with PRO data improves consistency in symptomatic AE assessment [22]. However, although providing additional insights into patients' experience, PROs data remain subjective and may be affected by missing data and challenges in interpretation [20]. Therefore, CTCAE and PROs are complementary rather than interchangeable tools, jointly contributing to a comprehensive understanding of AE trajectories and impact of treatment. The increasing use of electronic PROs offers opportunities for real-time symptom monitoring, although efforts are needed to address barriers in implementation and equitable access [23].

Disagreement between clinicians and patients is particularly evident for lower-grade AEs, which are often more subjective and challenging to assess consistently [9]. This was observed in Case 1, where AEs such as *Abdominal pain* and *Flatulence* showed variability in grading, partly due to broad and non-specific CTCAE definitions. Mild and moderate AEs should not be overlooked, as growing evidence suggests they contribute to overall patient burden, may lead to treatment discontinuation, and, when persistent, can affect long-term QoL [6], [9], [24], [25]. Consequently, these events are clinically meaningful and should be captured in clinical trials [26]. Furthermore, evaluation of the impact of treatment requires more than the reporting of AE severity alone [27], [28], as dimensions such as timing, duration, and frequency also play a role. However, these aspects remain insufficiently reported despite their relevance [8], [24], [29].

The greater focus on low-grade AEs introduces challenges related to data volume, management, and resources. While CTCAE terms are mapped to MedDRA to promote standardized reporting, inconsistencies in appropriate term selection remain problematic. The use of different MedDRA terms to describe the same clinical condition can reduce reliability and comparability, as illustrated in Case 2, where rectovaginal fistula led to different grading despite being described by the same definition. Such discrepancies highlight how the complexity of modern trials can limit consistent application and compromise data interpretation. Therefore, a balance is required between comprehensive AE documentation and practical feasibility. Selective data collection and reduction of redundant or synonymous terms may improve reporting efficiency and, in the meantime, help to avoid the generation of large volumes of uninformative data. Emphasis should be placed on clinically meaningful events, supported by training and pre-selection of preferred terms [6], [30], [31].

Given the importance of training, the interactive workshop was freely accessible and made available on demand. However, some limitations should be acknowledged. Although based on realistic scenarios, the clinical cases did not fully capture the complexity of patients' experiences, as the information remained filtered through the interpretation of the presenting clinician. The narrative format further limited the inclusion of patient perspectives. Moreover, the allocation of 40 min for two cases limited the depth of the information presented. Finally, participation in the live poll was limited (approximately 40% of attendees), limiting the ability to perform subgroup analyses, including by professional experience. This may be attributable to the heterogeneous live audience composition, including medical physicists and other non-medical professionals who may have lacked confidence in AE assessment.

In conclusion, BRIDGE is a community-driven initiative that places patient experience at the centre of AE reporting, extending beyond clinical trial applications. Accurate AE documentation is essential not only for evaluating treatment safety but also for identifying outcomes that matter most to patients and defining meaningful endpoints. Understanding symptom burden and interventions supports more effective and holistic supportive care through multidisciplinary collaboration and active patient involvement. The international participation in the webinar series supports the relevance of this topic, and BRIDGE may serve as a model for promoting similar initiatives across other tumour sites.

## CRediT authorship contribution statement

**Sofia Spampinato:** Writing – original draft, Visualization, Supervision, Project administration, Methodology, Investigation, Formal analysis, Conceptualization. **Ewa Burchardt:** Writing – review & editing, Resources. **Magdalena Stankiewicz:** Writing – review & editing, Resources. **Amelia Barcellini:** Writing – review & editing, Resources. **Gemma Eminowicz:** Writing – review & editing, Resources. **Barbara Šegedin:** Writing – review & editing, Resources. **Margit Valgma:** Writing – review & editing. **Kari Tanderup:** Writing – review & editing, Conceptualization. **Remi Nout:** Writing – review & editing, Conceptualization. **Kathrin Kirchheiner:** Writing – review & editing, Methodology, Conceptualization.

## Declaration of competing interest

The authors declare that they have no known competing financial interests or personal relationships that could have appeared to influence the work reported in this article. RN declares research grants paid to the institution from Elekta, Varian, Siemens Healthineers, Accuray, Sensius, Dr. Sennewald, and MSD, and speaker honoraria paid to the institution from Elekta, GSK, and MSD.

## Appendix A Supplementary data

Supplementary material Information on Clinical case 1 and Clinical case 2.
