# Supplementary material for: Adverse events in gynaecological radiotherapy: Better Reporting, Interpretation and Dissemination to Generate Evidence (BRIDGE)
Source: Tech Innov Patient Support Radiat Oncol. 2026 Sep 27;40:100439. doi: 10.1016/j.tipsro.2026.100439 (PMC13661216; doi:10.1016/j.tipsro.2026.100439)
Supplement: Supplementary file 1 [file mmc1.pdf]

Case 1

Baseline information

- **Age:** 40 years old
- **Previous surgery:** vaginal and perineal plastic surgery performed two years before oncological treatment
- **Smoking history:** Smoker
- **Diagnosis:** Cervical squamous cell carcinoma
- **Stage:** FIGO IIB

Primary treatment

- EBRT: 45 Gy in 25 fractions.
- Concurrent cisplatin ×5 cycles.
- Brachytherapy: 4 fractions of 7 Gy.

9 months after treatment completion

The patient developed progressive gastrointestinal symptoms, including alternating constipation and diarrhea, abdominal distension, flatulence, and recurrent abdominal pain. Loose stools occurred up to five to six times daily, while episodes of constipation could persist for several days. Despite treatment with antispasmodic agents, symptoms continued to worsen. The patient also reported lower back pain, fatigue, sleep disturbances, and a substantial decline in quality of life, ultimately preventing her from returning to work.

20 months after treatment completion

The patient experienced recurrent exacerbations of abdominal symptoms: One severe episode occurred after the consumption of raw apples, resulting in intense abdominal pain that required emergency department evaluation. Due to symptom severity, hospitalization was necessary on two occasions.

Diagnostic investigations, including abdominal ultrasonography and computed tomography, demonstrated marked gaseous distension of the transverse colon without evidence of mechanical bowel obstruction. The clinical presentation was attributed to functional bowel dysfunction characterized by excessive spasticity of the descending and sigmoid colon. These abnormalities resulted in recurrent constipation, abdominal distension, and pain predominantly localized to the right side of the abdomen.

Symptomatic improvement was achieved during hospitalizations following intravenous treatment. The patient received antispasmodic medications, metamizole, sodium butyrate supplementation, and probiotics, which provided temporary relief. However, recurrent episodes of gastrointestinal dysfunction persisted, prompting further evaluation for late radiation-induced bowel toxicity.

In addition to gastrointestinal complications, the patient experienced several long-term treatment-related consequences that significantly affected her overall well-being and daily functioning. These included post-treatment weight gain , persistent low mood, and depressive symptoms (06-11.2023). Together with chronic pain, fatigue, and sleep disturbances, these sequelae contributed substantially to reduced quality of life and prolonged occupational disability.

Case 2

Baseline information

- **Age:** 58 years old
- **Performance status:** ECOG 0
- **Comorbidities:** None reported.
- **Smoking history:** Never smoker
- **Diagnosis:** Cervical squamous cell carcinoma, Grade 1
- **Stage:** FIGO IIIB
- **Baseline imaging:**
  - MRI: Extensive cervical tumour involving the uterine corpus, right parametrium and vagina, suspected bladder and rectal wall infiltration; pelvic nodes suspicious for metastases.
  - CT: Extensive cervical tumour with uterine corpus and likely right parametria involvement, possible bladder and rectal invasion, and suspicious pelvic lymph nodes.

Primary treatment

- EBRT: 50.4 Gy in 28 fractions.
- Concurrent cisplatin ×6 cycles.
- Brachytherapy: 50 Gy in 5 fractions prescribed 1 cm from applicator axis.

Recurrence (20 months after primary treatment)

- MRI: Radiologically suspicious lesion in the right parametrium (37×37×38 mm). The lesion closely abuts and externally involves the anterior rectal wall.

First re-treatment (21 months after primary treatment)

- SBRT re-irradiation: 10 Gy in 2 fractions.

Locoregional progression (27 months after primary treatment)

- CT: A heterogenous solid-cystic mass (54×35×51 mm) in the right posterior uterus, extending from the cervix to the fundus. Possible focal rectal wall disruption adjacent to the tumour, with concern for fistula formation. Enlarged inguinal nodes compared.

Second re-treatment (28–30 months after primary treatment)

- Chemotherapy: 3 cycles of carboplatin and paclitaxel

Case 2

Follow-up after primary treatment	Event	Clinical description
20 months	Vaginal stenosis:	On physical examination, the vaginal canal was short and narrow, with obliteration of the upper part.
23 months	Progressive pelvic necrosis / threatened fistula	MRI demonstrated extensive tumour involvement with post-radiotherapy necrotic and inflammatory changes, increasing necrosis of the involved rectal wall and threatened fistula formation; RECIST: stable disease
24 months	Diarrhoea	Patient reported regular bowel movements with mildly loose stools.
26 months	Rectovaginal / uterorectal fistula	MRI showed extensive necrotic and inflammatory changes with suspected fistulous communication between the uterine cavity and rectum. RECIST: stable disease.<br>The patient also reported foul-smelling vaginal discharge.
26 months	Fatigue	Weakness; the patient remained independent in personal care, but had stopped performing routine household chores.
27 months	Urinary tract obstruction	Right hydronephrosis requiring insertion of a double-J ureteral stent. Laboratory tests showed creatinine 116 μmol/L and eGFR 44 mL/min/1.73 m^2.
30 months	Rectovaginal fistula	Passage of gas and small amounts of stool through the vagina. On examination, a ~3 mm fistula was identified at the right vaginal fornix–wall junction.
30 months	Peripheral sensory neuropathy	Paresthesia of the lower limbs; neurological examination showed normal gait and no motor deficits.
31 months	Diverting stoma	Elective stoma creation performed because of the symptomatic fistula.
34 months	Anorexia	Decreased appetite, while oral intake remained unchanged and body weight was stable.
